# Cryopyrin-associated periodic syndromes (CAPS): report on two cases (Muckle-Wells syndrome (MWS) and CINCA/NOMID)

**DOI:** 10.1186/1546-0096-9-S1-P3

**Published:** 2011-09-14

**Authors:** SO Salugina, ES Fedorov, NN Kuzmina

**Affiliations:** 1 Institute of Rheumatology RAMS, Moscow, Russia

## Background

Cryopyrin-associated periodic syndromes (CAPS) are the rare hereditary autoinflammatory diseases. CAPS include three similar conditions are distinguished which lie along a phenotypical continuum with increasing levels of severity: familial cold autoinflammatory syndrome (FCAS), Muckle-Wells syndrome (MWS) and CINCA/NOMID. Distinguishing features include cutaneous, neurological, ophthalmologic and rheumatologic manifestations. CAPS results from a gain-of-function mutation of the NLRP3 gene (CIAS1) coding for cryopyrin. There is no production of autoantibodies, but interleukin-1 plays an important role and acute-phase reactants show abnormalities.

Our aim was to report on two cases of CAPS that are considered to be rare entities.

## Case reports

In our clinic we observed two cases: MWS and CINCA/NOMID.

### The case 1

A 16-year-old Caucasian female had had symptoms beginning at the age of one year, including fever, recurrent urticaria, conjunctivitis, localized abdominal pain, neutrophil leukocytosis, since the 12 years periodic arthritis, which lasted a few days, otitis, uveitis. The laboratory findings included an ESR of up to 62 mm/h, anemia, neutrophil leukocytosis, C-reactive proteine rise, ANA and RF were negative. 15 years after the symptoms began, sensorineural deafness was diagnosed and MWS was suspected. A genetic analysis on the patient showed a new mutation (heterozygous) in the CIAS1 gene (c.1049C>T), thus confirming the diagnosis. The patient was treated with antibiotics and methotrexate without any response.

### The case 2

An 10-month-old boy complained of daily fever, recurrent since the first days of a life, blepharitis and conjunctivitis, neuroretinitis, panuveitis, sensorineural deafness, arthralgia, mixed hydrocephaly with the signs of cortex, visual nerves and corpus collosum atrophy on brain MRI. The laboratory findings included an ESR of up to 40 mm/h, anemia, neutrophil leukocytosis, C-reactive proteine rise, ANA and RF were negative, Interleukin-1- 152 pg\ml (under 5,0). With these findings, CINCA syndrome was suspected. A genetic analysis on the patient showed a new mutation in the CIAS1 gene (G569R), thus confirming the diagnosis. The patient was treated with prednisone with a partial response. Since beginning treatment with anti-interleukin-1(anakinra) up to 4mg/kg/d pts has had excellent control of his disorder.

## Conclusions

We report two rare cases of CAPS. These pts presented fever, cutaneous, neurological, ophthalmologic and rheumatologic manifestations, that dramatically improved with anakinra in one of pts. Pts with CAPS need anti IL-1 treatment for the successful in suppressing inflammation and reduction in the number and duration attacks.

